# Direct pars repair techniques for lumbar spondylolysis: a comparative literature review

**DOI:** 10.55730/1300-0144.6338

**Published:** 2026-04-28

**Authors:** Kamil Çağrı KÖSE, Moustafa IBRAHIM, Nicholas Donald HADEN, John AFOLAYAN, Hanny ANWAR

**Affiliations:** Department of Spinal Surgery, Royal National Orthopaedic Hospital Stanmore, Brockley Hill, Stanmore, United Kingdom

**Keywords:** Buck technique, Scott wiring, Morscher’s hook-screw, V-rod technique, smiley face construct, dual-stability construct

## Abstract

**Background/aim:**

Lumbar spondylolysis, a pars interarticularis defect affecting approximately 3%–6% of the general population, presents a significant clinical challenge in young adults and athletes. When conservative management fails, direct pars repair offers an attractive alternative to spinal fusion by preserving segmental motion and reducing the risk of adjacent segment degeneration.

This narrative review compares seven principal direct pars repair techniques—Buck’s technique, Scott’s wiring method, Morscher’s hook-screw system, pedicle screw-based constructs, the V-rod/U-rod (Gillet technique), the smiley face rod construct, and the dual-stability construct (DSC)—evaluating their biomechanical properties, clinical outcomes, fusion rates, and complication profiles.

**Materials and methods:**

A narrative literature review was conducted examining metaanalyses, retrospective cohort studies, and biomechanical investigations published between 1970 and 2024. Primary outcome measures included fusion rates, complication rates, clinical success rates, and biomechanical stability. The inherent heterogeneity of included studies—encompassing varied patient populations, surgical technique modifications, follow-up durations, and outcome definitions—precludes formal metaanalytic synthesis and is addressed in the limitations section.

**Results:**

Pedicle screw-based techniques demonstrated the highest fusion rates (90.2%; N = 240, 14 studies) with complication rates of 12.8% and positive clinical outcomes in 80.1% of patients. Buck’s technique showed fusion rates of 83.5% (N = 372, 27 studies) with complications in 13.4% and favourable outcomes in 84.3%. Scott’s wiring achieved 81.6% fusion with 22.4% complications. Morscher’s hook-screw system demonstrated 77.7% fusion rates (N = 193, five studies) with the highest complication rate at 27.4%. Modern constructs—the V-rod/U-rod, smiley face rod, and DSC—appear to offer comparable or superior outcomes to pedicle screw constructs in selected series. Age below 25 years emerged as the most critical determinant of success across all techniques.

**Conclusion:**

Pedicle screw-based direct pars repair appears to provide the most favourable fusion and complication profile among the classical techniques. Modern constructs (V-rod, smiley face, and DSC) show promising results and warrant prospective evaluation. Buck’s technique remains a viable alternative in experienced hands. Morscher’s hook-screw system should be reserved as a salvage option when pedicle screw trajectory is anatomically precluded. Patient selection criteria—including age below 45 years, minimal disc degeneration, and absence of significant spondylolisthesis—are central to surgical success.

## 1. Introduction

Lumbar spondylolysis represents a stress-induced fracture of the pars interarticularis, predominantly affecting the L5 vertebra in 68%–85% of cases [1,2]. The condition arises from repetitive mechanical stress, particularly in athletes engaged in hyperextension activities such as gymnastics, weight lifting, and cricket [3,4]. The incidence in the general population ranges from 3% to 6%, with higher rates observed in specific athletic populations [5]. While the majority of patients respond favourably to conservative treatment, approximately 5%–10% require surgical intervention due to persistent, disabling symptoms [2,6].

Posterolateral fusion was historically the standard surgical approach [7]. This technique sacrifices segmental motion and increases the risk of adjacent segment disease—a consequence particularly concerning in young patients with decades of functional life expectancy [8]. Direct pars repair emerged as a motion-preserving alternative, aiming to restore anatomical integrity of the pars interarticularis while maintaining segmental function [9,10].

Since Kimura’s pioneering work in 1968 introducing bone grafting without internal fixation [11], numerous techniques have evolved to enhance stability and fusion rates. The development of Buck’s screw fixation in 1970 [12], Scott’s tension-band wiring in 1986 [13], Morscher’s hook-screw system in 1984 [14], and pedicle screw-based constructs from the mid-1990s [15,16] expanded the surgical armamentarium. More recently, the V-rod/U-rod technique described by Gillet and Petit in 1999 [49], the smiley face rod construct, and the dual-stability construct (DSC) have further diversified available options. Despite this proliferation, consensus regarding the optimal surgical approach remains elusive [9,17].

This narrative review provides a comparative evaluation of all seven principal direct pars repair methodologies, analysing their biomechanical foundations, clinical efficacy, and contemporary relevance.

## 2. Materials and methods

### 2.1. Review type and search strategy

This manuscript constitutes a narrative literature review. It does not employ formal PRISMA methodology, prespecified inclusion algorithms, or risk-of-bias assessment tools, given the heterogeneous nature of the available literature and the descriptive, technique-oriented objectives of the review.

A comprehensive literature search was conducted using the PubMed, Cochrane Library, Web of Science, and CINAHL databases. Search terms included ‘spondylolysis,’ ‘direct pars repair,’ ‘Buck technique,’ ‘Scott wiring,’ ‘Morscher hook-screw,’ ‘pedicle screw fixation,’ ‘V-rod spondylolysis,’ ‘smiley face rod construct,’ and ‘dual-stability construct.’ Studies published between 1970 and 2024 were included (Table 1).

### 2.2. Inclusion criteria

- Studies involving patients with symptomatic lumbar spondylolysis or grade I spondylolisthesis
- Surgical management via direct pars repair
- Minimum follow-up of 6 months
- Clearly defined outcome measures including fusion rates, complications, and clinical outcomes

### 2.3. Outcome measures

Primary outcomes included radiographic fusion rates assessed by computed tomography or plain radiographs, complication rates, and clinical outcomes measured by MacNab criteria, visual analogue scale (VAS), and Oswestry Disability Index (ODI). Secondary outcomes included operative time, blood loss, and return to activity timelines.

### 2.4. Limitations of available evidence

The reviewed literature is characterised by significant heterogeneity. Patient populations vary with respect to age, activity level, prior surgical history, and spondylolisthesis grade. Surgical technique modifications within each named category complicate direct comparisons. Follow-up durations range from 6 months to over 11 years and outcome definitions differ between studies. The predominance of retrospective designs and small sample sizes limits evidence strength, while publication bias may inflate success rates. No randomised controlled trials comparing techniques were identified. Evidence for the V-rod, smiley face, and DSC derives predominantly from case series and short-to-medium term follow-up studies; the four classical techniques are supported by larger pooled analyses. Differences in reported outcomes should therefore be interpreted as directional trends rather than statistically validated conclusions.

## 3. Surgical techniques

### 3.1. Buck’s technique

#### 3.1.1. Technical description

Buck’s technique, introduced in 1970, involves placement of a lag screw directly across the pars defect from the inferior aspect of the lamina to the superior articular process [12]. The procedure requires a precise screw trajectory to achieve compression across the fracture site. Entry points are typically created 10 mm lateral to the spinous process base at the inferior laminar edge. An autogenous bone graft, often harvested from the posterior iliac crest, is impacted into the debrided defect to promote biological fusion [18].

#### 3.1.2. Biomechanical properties

Buck’s technique effectively resists forces in flexion, extension, and rotation [19,20]. The direct lag screw compression creates a favourable environment for bone healing. Finite element analysis reveals relatively concentrated stress on the intervertebral disc under axial rotation compared to intersegmental fixation methods [20]. Cadaveric studies confirm that Buck screws restore stability at the involved segment and reduce abnormal disc stress patterns [21,22].

#### 3.1.3. Clinical outcomes

Metaanalysis incorporating 27 studies and 372 adult patients demonstrates fusion rates of 83.5% [17]. Complication rates approximate 13.4%, substantially lower than those of wire-based techniques [17,23]. Positive clinical outcomes occur in 84.3% of patients [17,18]. Age significantly influences results; patients under 25 years achieve success rates approaching 90% [9,18]. Mean surgical times range from 45 to 75 min with blood loss of 50–140 mL [18].

#### 3.1.4. Technical considerations

Buck’s technique demands considerable surgical expertise owing to the challenging screw trajectory. Nerve root injury risk exists, particularly in dysplastic anatomy [10]. Navigation and neuromonitoring enhance safety [24]. Minimally invasive percutaneous approaches maintain comparable fusion rates with reduced soft tissue trauma [25–27].

### 3.2. Scott’s wiring technique

#### 3.2.1. Technical description

Scott’s wiring technique employs sublaminar passage of 18-gauge stainless steel wire around the transverse processes bilaterally, with subsequent tightening around the inferior spinous process [13,28]. This creates a tension-band construct stabilising the pars defect. Modified versions incorporate pedicle screws to enhance stability, combining a wire tension-band with three-point fixation [29,30].

#### 3.2.2. Biomechanical properties

Scott’s technique provides less rigid fixation than screw-based methods [19]. Under flexion-extension loading, the wiring construct demonstrates inferior rotational stability relative to screw-rod-hook and Buck techniques [19,20]. Modified pedicle-screw-augmented constructs significantly improve stability, though even these permit greater range of motion at the repair site than rigid alternatives [30,32].

#### 3.2.3. Clinical outcomes

Long-term follow-up studies report success rates of 86% in patients younger than 25 years [28,30]. Fusion rates approximate 81.6% with complication rates of 22.4%—nearly double those of Buck’s technique [17,28]. Positive clinical outcomes occur in 82.5% of appropriately selected patients [17,28]. Grade I spondylolisthesis does not preclude successful repair when disc integrity is preserved [33].

#### 3.2.4. Complications

Sublaminar wire passage poses risks of nerve root injury, dural tears, and haemorrhage [28,29]. Wire breakage occurs in a subset of patients, typically after successful fusion [28]. Cable-screw construct modifications mitigate wire-related complications [32]. Return to athletic activity typically occurs within 6–8 months [30,34].

### 3.3. Morscher’s hook-screw system

#### 3.3.1. Technical description

Morscher’s technique utilises a specialised hook-screw linking the vertebral arch to the superior articular process, bypassing rather than crossing the pars defect [14]. The hook engages the superior articular process while the screw anchors into the lamina. A compression spring maintains consistent force across the healing defect [14,35,36].

#### 3.3.2. Biomechanical properties

The hook-screw construct provides fixation without hardware through the fracture site, theoretically reducing stress concentration in healing bone [14]. Comparative biomechanical studies demonstrate inferior stability relative to pedicle screw-based methods and Buck’s technique [19,20], with greater micromotion permitted at the repair site [20].

#### 3.3.3. Clinical outcomes

Fusion rates average 77.7% across 193 patients in five studies [17,35]. Positive clinical outcomes occur in 80.3% of patients, with pain relief in 79% at a mean follow-up of 3.5–11 years [17,35]. Complication rates of 27.4% are the highest among the major repair techniques, driven primarily by hook-related hardware loosening in up to 57% of patients in some series [17,35].

#### 3.3.4. Contemporary role

Given its inferior outcomes relative to modern constructs, Morscher’s hook-screw system should not be regarded as a primary technique. It is appropriately reserved as a salvage option when both pedicle screw and Buck’s trajectories are anatomically precluded. In such cases, thorough informed consent regarding the elevated complication profile and navigation assistance are essential [14,37].

### 3.4. Pedicle screw-based techniques

#### 3.4.1. Technical description

Pedicle screw-based techniques utilise bilateral pedicle screws connected via rods to laminar hooks or additional screws, creating rigid three-column fixation [15,16,38]. Standard or percutaneous placement is employed depending on the surgical approach, with autogenous or allograft bone impacted into the prepared defect [38,42].

#### 3.4.2. Biomechanical properties

Pedicle screw-based constructs provide the most rigid stabilisation among the direct repair techniques [19,20]. Finite element analysis and cadaveric studies confirm superior stability under all loading conditions [20,31]. Three-column fixation restricts motion at the repair site while distributing stress more evenly across the construct [16].

#### 3.4.3. Clinical outcomes

Metaanalysis of 14 studies with 240 patients demonstrates 90.2% fusion, 12.8% complications, and favourable outcomes in 80.1% [17]. Multiple series report healing rates approaching 100% at 6-month follow-up when appropriate selection criteria are applied [39,42]. Return to sports averages 5–6 months—earlier than with wire-based methods [34,45,46].

#### 3.4.4. Surgical considerations

Mean operative times range from 90 to 120 min with potentially greater blood loss than simpler techniques [38,42]. Navigation and robotic assistance enhance screw placement accuracy [24,47]. Hardware burden and potential adjacent segment stress elevation are the principal disadvantages, though clinical significance remains debatable given the superior fusion rates [22,42].

### 3.5. V-rod/U-rod (Gillet technique)

Originally described by Gillet and Petit in 1999, the V-rod technique involves bilateral pedicle screws connected by a contoured V- or U-shaped rod that applies compression directly across the pars defect [49]. The rod geometry provides reliable stabilisation in challenging anatomical configurations without requiring laminar hooks, thereby eliminating hook-related failure modes. A minimum 10-year follow-up series demonstrated satisfactory outcomes with high fusion rates and an acceptable complication profile, supporting its viability as a durable fixation option [49]. Reported fusion rates approximate 85%–90% in available series with complication rates comparable to those of pedicle screw-based constructs (~12%–15%). Age-stratified outcomes follow a pattern consistent with other pedicle-based techniques, with the best results in patients under 25 years.

### 3.6. Smiley face rod construct

The smiley face rod construct employs pedicle screws connected by a custom-contoured rod whose geometry approximates a curved smile configuration, distributing compressive forces across the pars defect with optimised rod angulation. Recent clinical series report fusion rates of approximately 87%–92% with a complication profile comparable to that of standard pedicle screw-rod-hook constructs (~11%–14%) [32]. The technique offers the biomechanical advantages of pedicle-based fixation with a rod geometry designed to maximise compressive force at the defect site. It is gaining recognition as a reproducible and biomechanically sound option, particularly in active younger patients in whom early return to sport is a priority. Mean operative times range from 80 to 110 min, depending on the approach.

### 3.7. Dual-stability construct (DSC)

The DSC represents a hybrid fixation philosophy combining rigid pedicle screw fixation with a dynamic stabilisation element. By modulating the mechanical environment at the defect site, the DSC aims to optimise the mechanobiological stimulus for bone healing [43,44]. Recent series indicate fusion rates of approximately 88%–93%—among the highest reported for direct pars repair—with complication rates of ~11%–13% [32,43,44]. The hybrid design may reduce adjacent segment stress compared to entirely rigid constructs, a potentially important advantage in young, active patients [43]. While long-term data remain limited, the DSC concept aligns with contemporary mechanobiology principles and represents a significant evolution in direct pars repair strategy.

## 4. Comparative analysis

### 4.1. Fusion rates

Among the classical techniques, fusion rates favour pedicle screw-based constructs (90.2%, N = 240, 14 studies), followed by Buck’s technique (83.5%, N = 372, 27 studies), Scott’s wiring (81.6%), and Morscher’s system (77.7%, N = 193, five studies) [17,23]. Modern constructs demonstrate competitive fusion rates: DSC approximately 88%–93%, smiley face rod 87%–92%, and V-rod 85%–90%, though these numbers derive from smaller case series rather than pooled metaanalyses. The clinically meaningful difference between techniques is most significant in older patients, where marginal differences in fusion rates carry greater consequence for long-term function (Table 2).

### 4.2. Complication profiles

Morscher’s hook-screw has the highest overall complication rate (27.4%), followed by Scott’s wiring (22.4%), Buck’s technique (13.4%), and pedicle screw-based constructs (12.8%) [17]. Modern constructs—V-rod, smiley face, and DSC—appear to have complication rates broadly comparable to those of pedicle screw-based techniques (~11%–15% in available series), without the wire-related complications of Scott’s method or hook-related failure modes of Morscher’s system (Table 3).

### 4.3. Clinical outcomes and return to activity

Clinical success rates show less variation than fusion rates: Buck’s technique leads at 84.3%, followed by Scott’s (82.5%), Morscher’s (80.3%), and pedicle screw constructs (80.1%) [17]. Modern constructs show broadly comparable clinical success (~82%–88%). Return to sport is fastest with pedicle screw-based and modern rigid constructs (5–6 months), intermediate with Buck’s technique (6–7 months), and slowest with wire-based and hook-screw methods (7–8 months) [30,34,45,46].

### 4.4. Age-stratified outcomes

Age is the most important prognostic factor across all techniques [6,9,17]. Patients under 25 years achieve success rates of 85%–100% regardless of technique, while those over 35 years demonstrate markedly inferior outcomes, particularly with wire-based and hook-screw methods. Modern constructs appear to attenuate age-related decline modestly compared to classical techniques, possibly due to superior biomechanical stability compensating for diminished biological healing capacity (Table 4).

### 4.5. Biomechanical hierarchy

Biomechanical rigidity ranks pedicle screw-based constructs highest, with DSC and smiley face rod constructs offering comparable stability via optimised geometry and hybrid designs [19,20,43,44]. The V-rod provides robust pedicle-based stability comparable to standard pedicle screw constructs [49]. Buck’s technique follows and then Morscher’s system, with Scott’s wiring demonstrating the least rigidity [19,20]. Construct rigidity during the critical early healing phase correlates positively with fusion success, explaining the performance hierarchy.

## 5. Patient selection and surgical planning

### 5.1. Ideal candidates

Ideal candidates for direct pars repair share specific characteristics: age 18–45 years, symptomatic spondylolysis or grade I spondylolisthesis, failure of conservative management for a minimum of 6 months, absence of significant disc degeneration (Pfirrmann grade <3), minimal facet arthropathy, positive pars infiltration test, and normal preoperative discography where indicated [6,9,10]. Athletes and active young adults represent the prototypical candidates, for whom motion preservation is paramount [4,34,46] (Table 5).

### 5.2. Contraindications

Relative contraindications include age exceeding 45 years, grade II or higher spondylolisthesis, significant disc degeneration, facet arthropathy, sagittal imbalance, dysplastic anatomy, and patient noncompliance [6,9,10]. Absolute contraindications encompass active infection, severe osteoporosis compromising fixation, and medical comorbidities precluding elective surgery [10]. A comprehensive preoperative assessment including MRI, CT, dynamic radiographs, and diagnostic pars infiltration guides appropriate patient selection and technique choice [9,48].

### 5.3. Technique selection algorithm

Technique selection integrates patient age, anatomical factors, surgeon experience, available implants, and resources [9,10]. For patients under 25 years with normal anatomy, pedicle screw-based techniques, Buck’s method, V-rod, or smiley face constructs all provide reliably favourable outcomes. In anatomically challenging cases in which pedicle screw trajectory is precluded, Morscher’s hook-screw may be considered as a last resort only, with thorough informed consent. Navigation assistance should be utilised wherever feasible [24,47] (Table 6).

## 6. Minimally invasive surgery, navigation, and robotics

The evolution toward minimally invasive approaches is a defining trend across all seven repair techniques [25,46]. Percutaneous pedicle screw placement, endoscopic visualisation, and tubular retraction systems reduce soft tissue trauma while maintaining construct integrity [26,27,40,41]. Early results suggest comparable fusion rates with reduced morbidity, shorter hospitalisation, and accelerated recovery [25,46]. V-rod, smiley face, and DSC constructs are increasingly performed via percutaneous or mini-open approaches, with construct geometry lending itself to fluoroscopy- or navigation-guided placement.

Navigation and robotic assistance facilitate minimally invasive execution by enhancing trajectory accuracy despite limited direct visualisation [24,47]. Robot-assisted intralaminar screw fixation has been described for spondylolysis in adolescents, with potential to reduce radiation exposure and improve precision [47]. Future studies should explicitly stratify outcomes by surgical approach (open versus MIS) and technology utilisation to enable meaningful comparisons.

## 7. Future directions

### 7.1. Biological augmentation

Incorporation of bone morphogenetic proteins, platelet-rich plasma, and other biological adjuvants aims to enhance fusion rates, particularly in challenging cases [50,51]. Concerns regarding cost, off-label use, and long-term efficacy mandate cautious adoption pending definitive evidence [50,51].

### 7.2. Patient-specific approaches

Three-dimensional printing facilitates custom instrumentation and preoperative trajectory planning [47]. Computational modelling may optimise construct selection by accounting for individual anatomic variation and biomechanical requirements [20,47].

### 7.3. Outcome standardisation and prospective research

Standardisation of outcome measures and longer-term follow-up are essential to refine understanding of technique-specific performance [6,23]. Randomised controlled trials comparing all seven techniques are urgently needed. Prospective registries capturing patient-reported outcomes, activity-specific function, and validated quality-of-life instruments will provide more robust comparative data than the predominantly retrospective literature currently available [6,42].

## 8. Limitations

This review has several important limitations. The predominance of retrospective designs introduces selection bias and limits causal inference. Sample sizes are small for most series, reducing statistical power and generalisability. Substantial heterogeneity exists across studies regarding patient age, activity level, technique modification, graft choice, follow-up duration, and outcome definitions, precluding formal statistical metaanalysis. No randomised controlled trials were identified. Publication bias likely inflates reported success rates. Historical series spanning five decades introduce confounding from evolving perioperative care and implant design. Evidence for V-rod, smiley face, and DSC derives from smaller, more recent series compared to the classical techniques; this reflects their more recent introduction rather than inferior efficacy, but the evidence base for definitive comparison remains limited.

## 9. Conclusions

Direct pars repair for symptomatic lumbar spondylolysis represents an effective motion-preserving alternative to spinal fusion in carefully selected patients [6,9,10,17]. Among the classical techniques, pedicle screw-based constructs appear to provide the most favourable fusion and complication profile, followed by Buck’s technique, Scott’s wiring, and Morscher’s hook-screw system [17,23]. Modern constructs—the V-rod/U-rod, smiley face rod, and DSC—show promising outcomes in available case series and appear to offer results comparable to or exceeding those of standard pedicle screw-based constructs, warranting prospective evaluation.

Pedicle screw-based repair may be considered the reference technique when expertise and resources permit [17,38,42]. Buck’s technique provides an acceptable alternative in experienced hands [17,18]. Morscher’s hook-screw system should be reserved as a salvage option when pedicle screw and Buck’s trajectories are anatomically precluded, given its significantly higher complication rate (27.4%) and lower fusion rate (77.7%) relative to modern alternatives. Patient selection remains paramount: age below 45 years, minimal disc degeneration, absence of significant spondylolisthesis, and positive diagnostic testing are the strongest predictors of success [6,9,10]. Randomised controlled trials and prospective registries are urgently needed to provide definitive guidance for technique selection.

**Order of authors (with contributor roles):**

1. Kamil Cagri Kose, M.D. (Conceptualisation: Lead; Investigation: Equal; Writing – original draft: Equal)
2. Moustafa Ibrahim, Spinal Fellow (Conceptualisation: Equal; Data curation: Equal; Formal analysis: Supporting; Methodology: Equal; Writing – review & editing: Equal)
3. Nicholas D. Haden (Investigation: Equal; Validation: Equal; Writing – review & editing: Equal)
4. John Afolayan (Conceptualisation: Equal; Investigation: Equal; Software: Equal; Validation: Lead; Writing – review & editing: Supporting)
5. Hanny Anwar (Conceptualisation: Equal; Formal analysis: Lead; Methodology: Lead; Writing – original draft: Equal; Writing – review & editing: Equal)

## Figures and Tables

**Table 1:** Overview of all seven direct pars repair techniques.

Technique	Year	Primary fixation	Hardware components	Key biomechanical principle
Buck’s technique	1970	Lag screw compression	Single lag screw across pars defect	Direct axial compression across fracture site promotes bone healing
Scott’s wiring	1986	Tension-band wiring	Sublaminar wire looped around transverse processes and spinous process	Dynamic tension-band generates compression across defect during motion
Morscher’s hook-screw	1984	Hook-screw construct	Hook engaging superior articular process; screw in lamina; compression spring	Indirect fixation bypassing defect; compression spring maintains force
Pedicle screw-based	1997	Three-column fixation	Bilateral pedicle screws, connecting rods, laminar hooks	Rigid intersegmental stabilisation; superior biomechanical stability
V-rod / U-rod (Gillet)	1999	V/U-shaped contoured rod	Bilateral pedicle screws connected by contoured V- or U-rod	Robust pedicle-based fixation; reliable in challenging pars anatomy
‘Smiley face’ rod construct	2010s	Contoured smile-shaped rod	Pedicle screws connected by custom-contoured ‘smile’ rod	Optimised rod geometry distributes compressive forces across defect
Dual-stability construct	Recent	Hybrid rigid-dynamic fixation	Combined rigid pedicle fixation with dynamic stabilisation element	Mechanobiological optimisation of healing environment; high fusion rates

DSC = Dual-stability construct.

* Outcomes for V-rod, smiley face rod, and DSC are derived from case series and individual cohort studies [32,43,44,49].

**Table 2:** Comparative outcomes across all seven direct pars repair techniques.

Technique	Studies (n)	Patients (N)	Fusion (%)	Complication (%)	Clinical success (%)	Op. time (min)	Return to sport (months)
Pedicle screw-based	14	240	90.2	12.8	80.1	90–120	5–6
Buck’s technique	27	372	83.5	13.4	84.3	45–75	6–7
Scott’s wiring	Variable	Variable	81.6	22.4	82.5	60–90	7–8
Morscher’s hook-screw	5	193	77.7	27.4	80.3	75–105	7–8
V-rod / U-rod (Gillet)	Case series	Variable	~85–90*	~12–15*	~83–88*	75–100	6–7
‘Smiley face’ rod	Case series	Variable	~87–92*	~11–14*	~82–87*	80–110	5–7
Dual-stability construct	Case series	Variable	~88–93*	~11–13*	~83–88*	90–120	5–7

* Outcomes for V-rod, smiley face rod, and DSC are estimated ranges derived from available case series and cohort data [32,43,44,49]; they are not derived from pooled metaanalysis.

Classical technique data from Mohammed et al. [17] and Tsai et al. [23].

**Table 3:** Complication profiles across all seven direct pars repair techniques.

Complication	Buck’s	Scott’s	Morscher’s	Pedicle Screw	V-Rod	Smiley Face	DSC
Overall Rate	13.4%	22.4%	27.4%	12.8%	~12–15%*	~11–14%*	~11–13%*
Hardware loosening	3–5%	5–8%	15–20%	2–4%	2–4%	2–4%	2–4%
Hardware breakage	1–2%	8–12%	3–5%	1–2%	1–2%	1–2%	1–2%
Nerve root injury	2–4%	4–6%	3–5%	2–3%	2–3%	2–3%	2–3%
Dural tear	1–2%	3–5%	2–3%	1–2%	1–2%	1–2%	1–2%
Wound infection	2–3%	2–4%	3–4%	1–3%	1–2%	1–2%	1–2%
Nonunion	16.5%	18.4%	22.3%	9.8%	~10–15%*	~8–13%*	~7–12%*
Graft site morbidity	3–5%	3–5%	4–6%	3–4%	3–4%	3–4%	3–4%
Reoperation	5–8%	8–12%	10–15%	4–6%	4–6%	3–5%	3–5%

* Complication estimates for V-rod, smiley face, and DSC are approximate ranges from available case series [32,43,44,49].

Classical technique data from references [10,17,28,35].

**Table 4:** Age-stratified success rates across all seven direct pars repair techniques (%).

Age group	Buck’s (%)	Scott’s (%)	Morscher’s (%)	Pedicle screw (%)	V-rod (%)	Smiley face (%)	DSC (%)
<20 years	90–95	88–92	85–90	92–100	88–94	90–95	91–96
20–25 years	85–90	82–88	75–82	88–95	85–90	86–91	87–92
25–35 years	75–82	65–75	60–70	82–88	78–84	79–85	80–86
35–45 years	60–70	45–60	40–55	70–80	65–74	66–75	67–76
>45 years	<50	<40	<35	55–65	50–60	50–60	50–60

* Data for classical techniques from references [9,17,18,28,30,35].

Numbers for V-rod, smiley face, and DSC are estimated from available case series [32,43,44,49] and extrapolated from mechanobiological principles; prospective age-stratified data for these techniques are lacking.

**Table 5:** Patient selection criteria for direct pars repair.

Category	Ideal candidates	Relative contraindications	Absolute contraindications
Age	18–45 years	>45 years	N/A
Slip grade	Grade 0–I	Grade II	Grade III or higher
Disc integrity	Pfirrmann grade <3	Pfirrmann grade 3–4	Pfirrmann grade >4
Facet joints	Minimal arthropathy	Moderate arthropathy	Severe arthropathy
Conservative Rx	Failed >6 months	<6 months trial	Refused trial
Bone quality	Normal bone density	Mild osteopenia	Severe osteoporosis
Anatomy	Normal pars anatomy	Dysplastic anatomy	Severe dysplasia with instability
Diagnostic test	Positive pars infiltration	Equivocal testing	Negative testing
Patient factors	Compliant, motivated	Uncertain compliance	Active infection, severe comorbidities

**Table 6:** Technique selection algorithm based on patient characteristics.

Patient profile	First-line recommendation	Alternative option	Special considerations
Age <25 years, normal anatomy	Pedicle screw-based OR Buck’s technique	V-Rod / Smiley Face rod (equally effective modern alternatives)	Excellent outcomes with any technique; surgeon experience and implant availability drive choice
Age 25–35 years, normal anatomy	Pedicle screw-based	Buck’s technique; V-Rod or DSC if available	Prefer higher fusion rates given diminished biological healing capacity
Age 35–45 years	Pedicle screw-based or DSC	Consider fusion if disc degeneration present	Higher failure risk; meticulous preoperative disc and facet assessment essential
Dysplastic anatomy — pedicle trajectory unfeasible	Pedicle screw-based where trajectory permits; V-Rod as first alternative	Morscher’s hook-screw (salvage only when all screw-based options precluded)	Morscher’s carries 27.4% complication rate; reserved as last resort. Thorough informed consent and navigation assistance mandatory
Grade I spondylolisthesis	Pedicle screw-based or DSC	Buck’s technique; V-Rod	Increased stability requirement; confirm disc integrity with MRI preoperatively
Athletes requiring rapid return	Pedicle screw-based MIS or Smiley Face rod MIS	Buck’s technique MIS; V-Rod MIS	Minimise soft tissue trauma; percutaneous approaches preferred; rigidity aids early mobilisation
Resource-limited settings	Buck’s technique	Scott’s wiring	Lower implant cost; weigh against higher revision risk vs. modern constructs
Revision surgery	Pedicle screw-based	Consider fusion	Previous hardware complicates direct repair; fusion may be preferable depending on disc status

MIS = Minimally invasive surgery; DSC = Dual-stability construct. Morscher’s hook-screw is designated a salvage option only—reserved for cases in which both pedicle screw-based constructs and Buck’s technique are anatomically precluded. Its 27.4% complication rate and 77.7% fusion rate mandate thorough informed consent.
